# Supplementary material for: Milk Authentication: Stable Isotope Composition of Hydrogen and Oxygen in Milks and Their Constituents
Source: Molecules. 2020 Sep 2;25(17):4000. doi: 10.3390/molecules25174000 (PMC7504733; doi:10.3390/molecules25174000)
Supplement: Supplementary file 1 [file molecules-25-04000-s001.pdf]

Table S1. Geographical information of the sampling location together with $\delta^{18}O_{milk}$, $\delta^{18}O_{casein}$ and $\delta^{2}H_{casein}$ values during the summer and winter in the year period from 2012 to 2015.

							$\delta^{18}O_{milk,water}$ (‰)								$\delta^{18}O_{casein}$ (‰)				$\delta^{2}H_{casein}$ (‰)			
Sam. no.	Sample location	Geo -region	Latitude	Longitude	Distance from the coast (km)	Altitude (m)	2012		2013		2014		2015		2013		2014		2013		2014	
							sum	win	sum	win	sum	win	sum	win	sum	win	sum	win	sum	win	sum	win
1	Bohinj	Alpine	46°17'36.4"N	13°54'40.5"E	64	592	n.d.	n.d.	-5.2	-7.6	n.d.	-7.1	-4.0	n.d.	11.3	10.5	10.3	10.8	-134	-114	-134	-135
2	Tolmin	Alpine	46°11'08.9"N	13°43'52.5"E	48	201	n.d.	n.d.	n.d.	-6.1	-3.2	-6.4	-1.8	n.d.	n.d.	12.3	12.0	11.8	n.d.	-122	-124	-126
3	Kamnik	Alpine	46°13'01.5"N	14°37'04.8"E	91	308	-1.9	-5.7	-5.8	-6.1	-5.3	-6.6	-5.6	-6.5	9.7	10.7	12.2	10.7	-124	-138	-141	-129
4	Ribnica na Pohorju	Alpine	46°32'09.1"N	15°16'05.5"E	153	600	-2.2	-6.4	-4.8	-6.8	-5.1	-7.2	-5.3	-7.2	11.9	10.1	12.5	10.9	-124	-128	-122	-119
5	Mozirje	Alpine	46°20'21.3"N	14°57'40.5"E	121	340	-1.1	-5.1	-3.7	-6.7	-4.2	-6.6	-4.9	-6.8	n.d.	n.d.	10.1	10.3	n.d.	n.d.	-146	-128
6	Sav. dolina	Alpine	46°15'57.3"N	15°07'13.4"E	127	276	n.d.	n.d.	-5.4	n.d.	-5.4	-7.3	-5.7	-7.0	9.5	n.d.	12.3	n.d.	-130	n.d.	-145	n.d.
7	Vitanje	Alpine	46°22'48.7"N	15°17'41.8"E	144	449	n.d.	-6.5	-5.2	-6.7	-5.0	-7.0	-4.1	-6.7	10.3	10.8	n.d.	10.6	-116	-123	n.d.	-127
8	Ponikva	Alpine	46°15'14.1"N	15°26'32.4"E	147	338	n.d.	n.d.	-5.0	n.d.	-5.4	-7.0	-5.0	-6.6	n.d.	n.d.	12.1	11.4	n.d.	n.d.	-134	-115
9	Prevalje	Alpine	46°32'56.5"N	14°55'19.1"E	134	322	n.d.	n.d.	-5.9	n.d.	-5.3	-7.4	-4.6	-7.2	9.9	n.d.	12.4	10.8	-141	n.d.	-150	-122
10	Slovenj Gradec	Alpine	46°30'28.0"N	15°04'37.2"E	138	413	n.d.	-7.1	-5.6	-7.3	-5.7	-7.2	-4.9	-7.2	n.d.	10.4	11.7	11.3	n.d.	-115	-140	-122
11	Vrh	Alpine	46°34'30.5"N	15°02'05.0"E	141	346	n.d.	n.d.	-5.7	n.d.	-5.6	-7.3	-5.3	-7.3	8.8	n.d.	13.0	11.2	-113	n.d.	-145	-134
12	Dravog.	Alpine	46°35'23.5"N	15°01'21.8"E	141	390	n.d.	-7.3	-5.8	-7.2	-5.2	-7.3	-5.1	-7.0	10.5	11.0	11.9	11.4	-118	-124	-148	-133
13	Vuzenica	Alpine	46°35'53.8"N	15°09'54.4"E	150	365	n.d.	n.d.	-6.0	n.d.	-5.3	-7.2	-4.6	-7.0	9.9	n.d.	12.4	10.6	-124	n.d.	-143	-132
14	Polzela	Alpine	46°16'50.4"N	15°04'29.9"E	124	292	n.d.	-5.9	n.d.	-6.9	n.d.	-6.9	-5.4	-7.0	n.d.	10.1	n.d.	11.7	n.d.	-133	n.d.	-124
15	Zavodnje	Alpine	46°25'29.8"N	15°01'02.9"E	129	642	n.d.	-7.0	n.d.	-6.8	n.d.	-7.1	-5.1	-6.8	n.d.	11.7	n.d.	12.0	n.d.	-112	n.d.	-120
16	Šmarje pri Jelšah	Alpine	46°14'08.6"N	15°31'30.9"E	153	234	n.d.	-6.6	n.d.	-6.8	n.d.	-7.2	-5.8	-6.7	n.d.	10.4	n.d.	11.6	n.d.	-121	n.d.	-124
17	Škale	Alpine	46°23'28.1"N	15°05'29.9"E	132	456	n.d.	-6.7	n.d.	-6.6	n.d.	-6.9	-5.0	-6.6	n.d.	10.8	n.d.	10.8	n.d.	-139	n.d.	-133
18	Planina	Alpine	46°06'17.2"N	15°24'17.4"E	140	561	n.d.	-7.3	n.d.	-6.5	n.d.	-7.3	-5.4	-8.7	n.d.	9.6	n.d.	11.1	n.d.	-129	n.d.	-135
19	Braslovče	Alpine	46°17'18.8"N	15°02'20.8"E	123	307	n.d.	-7.3	n.d.	-6.8	n.d.	-7.1	-5.1	-8.8	n.d.	10.4	n.d.	11.2	n.d.	-127	n.d.	-139
20	Šmihel	Alpine	46°09'32.1"N	15°13'27.8"E	129	428	n.d.	n.d.	n.d.	n.d.	n.d.	-6.8	-5.8	-8.6	n.d.	n.d.	n.d.	11.7	n.d.	n.d.	n.d.	-127
21	Vinica	Dinaric	45°27'41.5"N	15°15'12.6"E	60	184	-3.3	-6.5	-5.5	-7.1	-5.4	-6.9	-5.5	-7.3	10.4	10.1	10.4	11.0	-130	-127	-138	-139
22	Velike Lašče	Dinaric	45°50'05.3"N	14°38'18.6"E	62	526	n.d.	n.d.	n.d.	n.d.	-5.2	-7.2	-5.4	-7.3	n.d.	n.d.	10.1	11.1	n.d.	n.d.	-146	-142
23	Kamenje	Dinaric	45°51'08.3"N	15°08'58.5"E	82	387	-3.6	-8.0	-6.3	-7.0	-5.5	-7.2	-5.6	-7.5	n.d.	n.d.	n.d.	n.d.	n.d.	n.d.	n.d.	n.d.
24	Logatec	Dinaric	45°55'01.6"N	14°13'32.7"E	48	476	-2.8	-5.8	-4.5	-7.0	-4.5	-6.8	n.d.	n.d.	10.7	10.9	11.9	10.6	-131	-126	-135	-132
25	Žužem.	Dinaric	45°49'53.4"N	14°55'41.3"E	70	209	-3.2	-6.8	-6.0	-7.5	-5.0	-6.9	-4.7	-7.8	10.5	9.2	12.0	n.d.	-115	-138	-120	n.d.
26	Podpeč	Dinaric	45°58'22.9"N	14°24'56.5"E	62	292	-3.4	-5.1	-5.1	-7.1	-5.4	-6.4	-5.8	-6.7	10.6	10.5	11.3	10.9	-126	-130	-127	-142
27	Postojna	Dinaric	45°46'29.4"N	14°12'55.7"E	39	556	-2.2	-6.3	-5.4	-6.0	-3.9	-6.2	-5.4	-6.1	12.8	9.1	12.6	11.9	-117	-120	-128	-112
28	Ribnica	Dinaric	45°45'6.97"N	14°42'59.64"E	79	492	-2.2	-7.5	-5.2	-7.0	n.d.	n.d.	n.d.	n.d.	10.1	9.7	n.d.	n.d.	-112	-120	n.d.	n.d.
29	Brkini	Mediterranean	45°34'17.8"N	14°02'35.8"E	20	588	-3.1	-4.7	-4.7	-5.6	-5.2	-7.0	-4.3	-6.1	10.7	10.7	12.5	11.6	-123	-114	-130	-120
30	Kozina	Mediterranean	45°36'30.0"N	13°55'43.9"E	11	493	-3.3	-3.6	-5.6	-5.1	-2.1	-4.4	-5.6	-4.5	9.8	9.8	14.6	12.6	-132	-112	-131	-111
31	Sežana	Mediterranean	45°42'09.1"N	13°51'06.5"E	10	360	-3.6	-6.7	-4.7	-7.7	-4.5	-6.9	-4.6	-8.0	9.6	n.d.	n.d.	n.d.	-121	n.d.	n.d.	n.d.

32	Godovič	Mediterranean	46°0'10.01"N	14°1'50.02"E	41	595	n.d.	-6.7	n.d.	n.d.	n.d.	n.d.	n.d.	n.d.	n.d.	n.d.	n.d.	n.d.	n.d.	n.d.	n.d.	n.d.
33	Vipava	Mediterranean	45°50′51.21″N	13°57′44.48″E	24	108	n.d.	-5.3	n.d.	n.d.	n.d.	n.d.	n.d.	n.d.	n.d.	n.d.	n.d.	n.d.	n.d.	n.d.	n.d.	n.d.
34	Zadlog	Mediterranean	45°57′11″N	14°00′59″E	34	715	0.3	n.d.	n.d.	n.d.	n.d.	n.d.	n.d.	n.d.	n.d.	n.d.	n.d.	n.d.	n.d.	n.d.	n.d.	n.d.
35	Brežice	Pannonian	45°55'17.1"N	15°35'50.1"E	149	162	-2.5	-7.0	-4.6	-6.5	-5.2	-7.4	-6.0	-7.1	9.7	10.2	10.8	9.8	-118	-131	-137	-134
36	Korena	Pannonian	46°31'27.1"N	15°46'20.1"E	188	350	-2.1	-5.9	-4.9	-6.3	-4.8	-7.2	-5.2	-7.1	11.2	10.0	n.d.	10.2	-123	-134	n.d.	-140
37	Ptuj	Pannonian	46°25'18.3"N	15°52'05.4"E	186	232	-2.7	-6.4	-5.5	-6.8	-5.6	-7.0	-5.7	-6.9	n.d.	n.d.	n.d.	11.4	n.d.	n.d.	n.d.	-120
38	Ptuj	Pannonian	46°25'18.3"N	15°52'05.4"E	186	232	-2.4	-6.6	-5.7	-6.4	-5.3	-6.9	-5.7	-6.9	9.7	10.8	12.7	n.d.	-129	-122	-133	n.d.
39	Ptuj	Pannonian	46°25'18.3"N	15°52'05.4"E	186	232	n.d.	-6.5	-5.3	-7.4	-6.5	-6.9	-6.0	-7.3	11.0	12.3	11.4	14.1	-116	-116	-144	-101
40	Rogaška Slatina	Pannonian	46°14'09.8"N	15°38'08.9"E	159	224	n.d.	n.d.	-5.5	n.d.	-5.3	-7.5	-5.5	-6.7	n.d.	n.d.	11.9	13.3	n.d.	n.d.	-141	-114
41	Slo. Konjice	Pannonian	46°20'16.1"N	15°25'23.6"E	152	322	n.d.	n.d.	-5.8	n.d.	-5.5	-7.0	-5.7	-6.5	10.9	n.d.	13.0	11.2	-117	n.d.	-140	-119
42	Z Goričko	Pannonian	46°49'27.6"N	16°05'09.1"E	222	270	n.d.	-6.5	-5.4	-6.9	-6.3	-7.0	-5.1	-7.1	10.3	11.1	11.3	n.d.	-142	-130	-130	n.d.
43	Ljutomer	Pannonian	46°32'09.9"N	16°11'54.8"E	214	175	n.d.	-6.4	-4.9	-6.9	-6.0	-6.9	-5.2	-7.2	10.7	9.4	13.0	14.4	-130	-116	-133	-100
44	Murska Sobota	Pannonian	46°39'29.4"N	16°09'47.5"E	218	189	n.d.	-6.8	-5.3	-6.5	-6.3	-7.0	-5.2	-6.9	12.6	11.1	10.9	13.8	-105	-122	-133	-104
45	Radenci	Pannonian	46°38'35.2"N	16°02'20.1"E	209	202	n.d.	-6.5	-4.7	-6.6	-6.3	-6.7	-4.9	n.d.	9.8	10.4	11.2	14.5	-115	-123	-137	-107
46	Ormož	Pannonian	46°25'10.7"N	16°08'46.2"E	203	218	n.d.	-6.7	-4.8	-7.5	-6.4	n.d.	n.d.	n.d.	9.3	10.4	11.9	n.d.	-114	-131	-134	n.d.
47	V Goričko	Pannonian	46°49'57.4"N	16°17'04.8"E	235	242	n.d.	-6.8	-5.3	-6.8	-6.0	-6.8	-5.6	-7.2	10.9	10.9	13.6	14.5	-113	-120	-121	-104
48	Gornja Radgona	Pannonian	46°40'38.7"N	15°59'23.5"E	207	206	n.d.	-6.5	-5.2	n.d.	-6.0	-7.2	-5.5	-7.0	10.2	11.6	12.0	13.7	-128	-110	-136	-122
49	Gornja Radgona	Pannonian	46°40'38.7"N	15°59'23.5"E	207	206	-6.0	-6.4	n.d.	n.d.	n.d.	n.d.	n.d.	n.d.	n.d.	n.d.	n.d.	n.d.	n.d.	n.d.	n.d.	n.d.
50	Sevnica	Pannonian	46°00'42.5"N	15°18'32.5"E	126	183	n.d.	-6.0	n.d.	-7.1	n.d.	-7.0	-5.6	-9.1	n.d.	9.0	n.d.	11.2	n.d.	-122	n.d.	-142
51	Maribor	Pannonian	46°32'50.5"N	15°38'42.8"E	176	262	n.d.	n.d.	n.d.	n.d.	n.d.	-7.2	-6.5	n.d.	n.d.	n.d.	n.d.	13.2	n.d.	n.d.	n.d.	-115
52	Šentilj	Pannonian	46°41'17.99"N	15°43'3.00"E	190	292	n.d.	-7.0	-6.0	-6.7	-5.5	-7.1	-5.2	-7.0	9.5	10.6	11.8	10.7	-149	-121	-138	-127
53	Slovenska Bistrica	Pannonian	46°23'34.01"N	15°34'27.98"E	162	275	n.d.	-6.8	n.d.	-6.7	n.d.	-7.4	-5.3	-7.0	n.d.	10.0	n.d.	n.d.	n.d.	-123	n.d.	n.d.
54	Lenart	Pannonian	46°33'28.76"N	15°48'49.79"E	190	264	n.d.	n.d.	n.d.	n.d.	n.d.	n.d.	n.d.	-7.3	n.d.	n.d.	n.d.	n.d.	n.d.	n.d.	n.d.	n.d.

Table S2. Data collection of the $\delta^{18}O$ values of milk of different dairy species: sheep and goat. For comparison, samples were collected during summer season from May to June in 2012 and 2013.

Location	Date sampling	Latitude	Longitude	Altitude (m)	Distance from the coast (km)	Species	$\delta^{18}O_{milk}$ (‰)
Branik	17.5.2012	45°51'4.32"N	13°47'30.48"E	87	19	sheep	-1.9
Ajdovščina	22.5.2012	45°53'9.64"N	13°54'34.06"E	106	25	sheep	-2.8
Gradac	29.5.2012	45°38'49.99"N	15°18'51.01"E	150	72	sheep	-1.6
Ilirska Bistrica	29.5.2012	45°34'43.61"N	14°18'23.47"E	421	26	sheep	1.2
Čičarija	30.5.2012	45°29'10"N	14°06'00"E	1028	25	sheep	0.1
Vremščica	23.5.2012	45°41′16″N	14°03′45″E	1027	22	sheep	-5.2
Vremščica	23.5.2012	45°41′16″N	14°03′45″E	1027	22	sheep	-5.6
Bovec	22.5.2012	46°20'15"N	13°33'10.01"E	454	64	sheep	-3.7
Log pod Mangartom	22.5.2012	46°24′21.27″N	13°36′11.29″E	644	69	sheep	-3.3
Soča	22.5.2012	46°20'36"N	13°39'23"E	492	63	sheep	-3.8
Bovec	22.5.2012	46°20'15"N	13°33'10.01"E	454	64	sheep	-2.1
Vransko	14.5.2012	46°14'38"N	14°57'5"E	340	116	sheep	-2.0
Senožete	28.5.2012	46°6′58.04″N	15°11′41.43″E	441	115	sheep	-2.8
Branik	8.6.2012	45°51'4.32"N	13°47'30.48"E	87	19	sheep	0.1
Ilirska Bistrica	15.6.2012	45°34'43.61"N	14°18'23.47"E	421	26	sheep	-0.7
Ilirska Bistrica	15.6.2012	45°34'43.61"N	14°18'23.47"E	421	26	sheep	-2.4
Ajdovščina	7.6.2012	45°53'9.64"N	13°54'34.06"E	106	25	sheep	-1.1
Soča	7.6.2012	46°20'36"N	13°39'23"E	492	63	sheep	-3.6
Bovec	12.6.2012	46°20'15"N	13°33'10.01"E	454	64	sheep	-3.6
Bovec	7.6.2012	46°20'15"N	13°33'10.01"E	454	64	sheep	-2.8
Ilirska Bistrica	15.6.2012	45°34'43.61"N	14°18'23.47"E	421	26	sheep	-0.7
Ilirska Bistrica	15.6.2012	45°34'43.61"N	14°18'23.47"E	421	26	sheep	-2.4
Ajdovščina	7.6.2012	45°53'9.64"N	13°54'34.06"E	106	25	sheep	-1.1
Soča	7.6.2012	46°20'36"N	13°39'23"E	492	63	sheep	-3.6
Bovec	12.6.2012	46°20'15"N	13°33'10.01"E	454	64	sheep	-3.6
Bovec	7.6.2012	46°20'15"N	13°33'10.01"E	454	64	sheep	-2.8
Dobrovo	24.5.2012	45°59'47"N	13°31'35"E	123	24	goat	-1.0
Renče	24.5.2012	45°53'24"N	13°40'07"E	51	13	goat	0.3

Miren	19.6.2012	45°53'44.02"N	13°36'27"E	50	13	goat	-2.0
Srpenica	26.5.2012	46°17'38"N	13°30'4"E	364	61	goat	-1.3
Miren	14.5.2012	45°53'44.02"N	13°36'27"E	50	13	goat	0.5
Miren	11.5.2012	45°53'44.02"N	13°36'27"E	50	13	goat	1.2
Cirkulane	23.5.2012	46°20′26″N	15°59′45″E	229	166	goat	-2.4
Laško	28.5.2012	46°09'16.67"N	15°14'7.98"E	260	116	goat	-5.3
Črni Kal	21.6.2012	45°33'16.18"N	13°52'9.79"E	253	8	goat	2.4
Srpenica	7.6.2012	46°17'38"N	13°30'4"E	364	61	goat	-0.4
Miren	19.6.2012	45°53'44.02"N	13°36'27"E	50	13	goat	-2.0
Bovec	13.5.2013	46°20'15"N	13°33'10.01"E	454	64	sheep	-4.4
Log pod Mangartom	13.5.2013	46°24′21.27″N	13°36′11.29″E	644	69	sheep	-4.7
Bovec	13.5.2013	46°20'15"N	13°33'10.01"E	454	64	sheep	-2.6
Bovec	13.5.2013	46°20'15"N	13°33'10.01"E	454	64	sheep	-4.2
Soča	13.5.2013	46°20'36"N	13°39'23"E	492	63	sheep	-4.1
Ajdovščina	13.5.2013	45°53'9.64"N	13°54'34.06"E	106	25	sheep	-2.6
Branik	13.5.2013	45°51'4.32"N	13°47'30.48"E	87	19	sheep	-2.9
Ajdovščina	13.5.2013	45°53'9.64"N	13°54'34.06"E	106	25	sheep	-3.6
Bovec	13.5.2013	46°20'15"N	13°33'10.01"E	454	64	sheep	-2.3
Črni Kal	22.5.2013	45°33'16.18"N	13°52'9.79"E	253	8	sheep	0.6
Čičarija	31.5.2013	45°29'10"N	14°06'00"E	1028	25	sheep	-5.0
Ilirska Bistrica	17.5.2013	45°34'43.61"N	14°18'23.47"E	421	26	sheep	-1.5
Srpenica	13.5.2013	46°17'38"N	13°30'4"E	364	61	goat	-1.6
Bovec	13.5.2013	46°20'15"N	13°33'10.01"E	454	64	goat	-0.8
Bovec	13.5.2013	46°20'15"N	13°33'10.01"E	454	64	goat	-0.8
Miren	13.5.2013	45°53'44.02"N	13°36'27"E	50	13	goat	-1.8
Renče	13.5.2013	45°53'24"N	13°40'07"E	51	13	goat	-1.8
Dobrovo	13.5.2013	45°59'47"N	13°31'35"E	123	24	goat	-1.9
Dobrovo	13.5.2013	45°59'47"N	13°31'35"E	123	24	goat	-3.9
